# Using Quantitative D-Dimer to Determine the Need for Pulmonary CT Angiography in COVID-19 Patients

**DOI:** 10.51894/001c.18652

**Published:** 2021-04-13

**Authors:** Gary Mikhjian, Ahmad Elghoroury, Keith Cronovich, Kevin Brody, Robert Jarski

**Affiliations:** 1 Emergency Medicine Henry Ford Macomb; 2 Family Medicine Henry Ford Macomb; 3 Emergency Medicine Henry Ford; 4 School of Health Sciences Oakland University

**Keywords:** ct angiography, d-dimer, pulmonary embolism, sars-cov-2, covid-19

## Abstract

**INTRODUCTION:**

COVID-19 has been frequently cited as a condition causing a pro-inflammatory state leading to hypercoagulopathy and increased risk for venous thromboembolism. This condition has thus prompted prior studies and screening models that utilize D-dimer for pulmonary embolism (PE) into question. The limited research to date has failed to provide tools or guidance regarding what COVID-19 positive patients should receive pulmonary CT angiography screening. This knowledge gap has led to missed diagnoses, CT overutilization, and increased morbidity and mortality.

**OBJECTIVE:**

The purpose of this study was to examine the utility of the quantitative D-dimer lab marker in a convenience sample of 426 COVID-19 positive patients to assist providers in determining the utility of pulmonary CT angiography.

**METHODS:**

The authors conducted a retrospective analysis on all COVID-19 positive patients within the Henry Ford Medical System between March 1st, 2020 through April 30th, 2020 who received pulmonary CT angiography and had a quantitative D-dimer lab drawn within 24 hours of CT imaging.

**RESULTS:**

Our sampling criteria yielded a total of n = 426 patients, of whom 347 (81.5%) were negative for PE and 79 (18.5%) were positive for PE. The average D-dimer in the negative PE group was 2.95 μg./mL. (SD 4.26), significantly different than the 9.15 μg./mL. (SD 6.80) positive PE group (P < 0.05; 95% CI -7.8, -4.6). Theoretically, applying the traditional ≤ 0.5 μg./mL. D-dimer cut-off to our data would yield a sensitivity of 100% and specificity of 7.49% for exclusion of PE. Based on these results, the authors would be able to increase the D-dimer threshold to < 0.89 μg./mL. to maintain their sensitivity to 100% and raise the specificity to 27.95%. Observing a D-dimer cut-off value of ≤ 1.28 μg./mL. would reduce sensitivity to 97.47% but increase the specificity to 57.93%.

**CONCLUSIONS:**

These study results support the utilization of alternative D-dimer thresholds to exclude PE in COVID-19 patients. Based on these findings, providers may be able to observe increased D-dimer cut-off values to reduce unnecessary pulmonary CT angiography scans.

## INTRODUCTION

In late 2019, a new strain of coronavirus known as severe acute respiratory syndrome coronavirus 2 (SARS-CoV-2) or “COVID-19” was discovered.^1^ The majority of the first COVID-19 cases were either asymptomatic or resulted in relatively mild disease with a broad range of symptoms.

During later 2020, thrombotic complications emerged as important sequelae contributing to significant morbidity and mortality in COVID-19 patients.^2^ More patients infected with COVID-19 may now be predisposed to thrombotic disease such as pulmonary embolism (PE) due to excessive inflammation, platelet activation, endothelial dysfunction, and stasis.^3^

In one 2020 US report of over 370,000 symptomatic confirmed COVID-19 patients, shortness of breath was cited to be present in 29% of cases.^4^ Similar to COVID-19, PE can have a wide range of presenting symptoms but the most common being shortness of breath.^5^ This overlap of symptoms and predisposition for thrombotic complications has posed a challenge for clinicians to promptly identify PE in COVID-19 patients. Rapid confirmation or exclusion of PE is vitally important for initiating appropriate therapy with anticoagulation.

Traditionally, the D-dimer lab marker (normally ≤ 0.5 μg./mL.) has been utilized in conjunction with clinical probability assessments to rule out PE.^6^ When elevated, the D-dimer should prompt further diagnostic imaging to identify PE, specifically, pulmonary CT angiography. Despite the increased risk of PE in COVID-19 patients, there remains no validation or consensus on D-dimer values and when to obtain pulmonary CT angiography.

### Purpose of Study

The purpose of this retrospective study was to examine the utility of quantitative D-dimer lab values in a convenience sample of confirmed COVID-19 patients to assist providers considering the need for pulmonary CT angiography to confirm or exclude PE.

## METHODS

After IRB project approval was obtained, the authors first conducted a retrospective analysis of all confirmed COVID-19 patients who received pulmonary CT angiography. Patients were further classified as being positive or negative for PE. Following this initial electronic health record (EHR) data extraction, the authors’ primary endpoint was to evaluate the quantitative D-dimer value between these two sample subgroups. Additional demographic and clinical patient information (e g., age, gender, presence of infiltrate on chest X-ray or CT, and mortality) were collected. Infiltrate was defined as an abnormal substance within the interstitium or alveoli of the lungs.

Study data were collected using the *EPIC Workbench EHR* from the Henry Ford Health System. An initial data report was generated by the Principal Investigator (GM) on May 15^th^, 2020 from all patients who tested positive for COVID-19 and received a pulmonary CT angiography during their ED visit or inpatient hospitalization. The authors included data points spanning from March 1^st^ through April 30^th^, 2020. This initially yielded 605 unique cases that were reduced to 426 patients after applying our inclusion and exclusion criteria.

Chart reviews by authors GM and AE of each eligible patient were utilized to ensure each patient was positive for COVID-19, had received pulmonary CT angiography, and had a documented D-dimer lab value obtained within 24 hours of the CT being performed. If a sample patient had received multiple (i.e., routinely ordered) D-dimer values, the D-dimer values that were drawn closest to the time the CT were completed was in analyses. In contrast, individuals who did not have a quantitative D-dimer within 24 hours were excluded. This approach was utilized by the authors to account for daily D-dimer values typically found in patients.

Furthermore, charts which were marked privacy restricted by EPIC (e g., prisoners and healthcare system employees) were excluded to maintain confidentiality. Finally, patients who underwent pulmonary CT angiography which were determined to be inadequate (i.e., unable to exclude PE) by the radiologist were excluded. Inadequate CT’s were most commonly attributed to poor image quality due to motion artifact (i.e., patient movement) and/or contrast bolus timing leading to poor contrast enhancement. However, CTs that were read as having a “limited evaluation” and still able to exclude pulmonary embolism to any degree were included.

### Data Analyses

All analyses were performed by author RJ using *Minitab Statistical Software* (State College, PA) or *Vassarstats.net* on-line calculator.^7,8^ Categorical data were summarized as counts and percentages, and continuous data were summarized as means with corresponding standard deviations. Between-group mean differences were compared by calculating t-tests for independent measures. Categorical data were compared using the chi-square test for association. Throughout this study, a p-value ≤ 0.05 (two-tailed) was considered statistically significant.

Sensitivities and specificities of D-dimer lab values for PE exclusion were calculated using different thresholds. Thresholds were chosen based on the distribution of our sample population data, with the overall goal of attempting to attain a PE sensitivity of 100%.

## RESULTS

### Sample Characteristics

**Figure 1.**
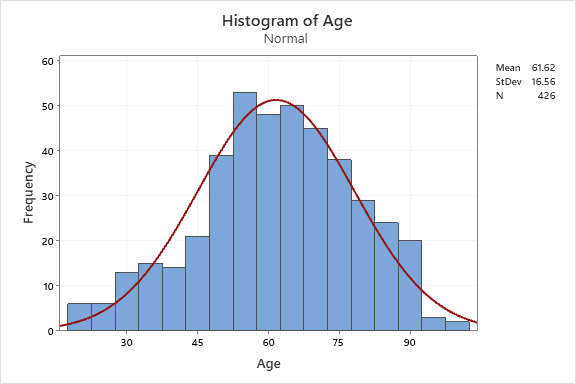
Age distribution of sample

Our sample population was gathered from the Henry Ford Health System between March 1^st^ through April 30^th^, 2020. After initial data extractions, a total sample of n = 426 (70.4%) patients were included in our analytic sample from an initial total of 605. The gender distribution of our sample included 209 males (49.1%) and 217 females (50.9%). The mean age in years for our sample population was 61.62 (SD 16.56) with a range of 19 to 99, further depicted in histogram form with superimposed normal distributional curve. (Figure 1).

### Main Results

Of the total 426 sample patients, 347 (81.5%) were negative for PE and 79 (18.5%) were positive for PE. The average D-dimer in the “negative for PE” group was 2.95 μg./mL. (SD 4.26) and significantly different compared to 9.15 μg/mL (SD 6.80) in the “positive for PE” group (P < 0.05) (Table 1).

**Table 1.** Main results comparing negative and positive pulmonary embolism subgroups.

**Group**	**N (%)**	**Average D-dimer (SD) (μg./mL.)**
**Negative for PE**	347 (81.5)	2.95 (4.26)
**Positive for PE**	79 (18.5)	9.15 (6.80)

PE = Pulmonary Embolism

**Figure 2.**
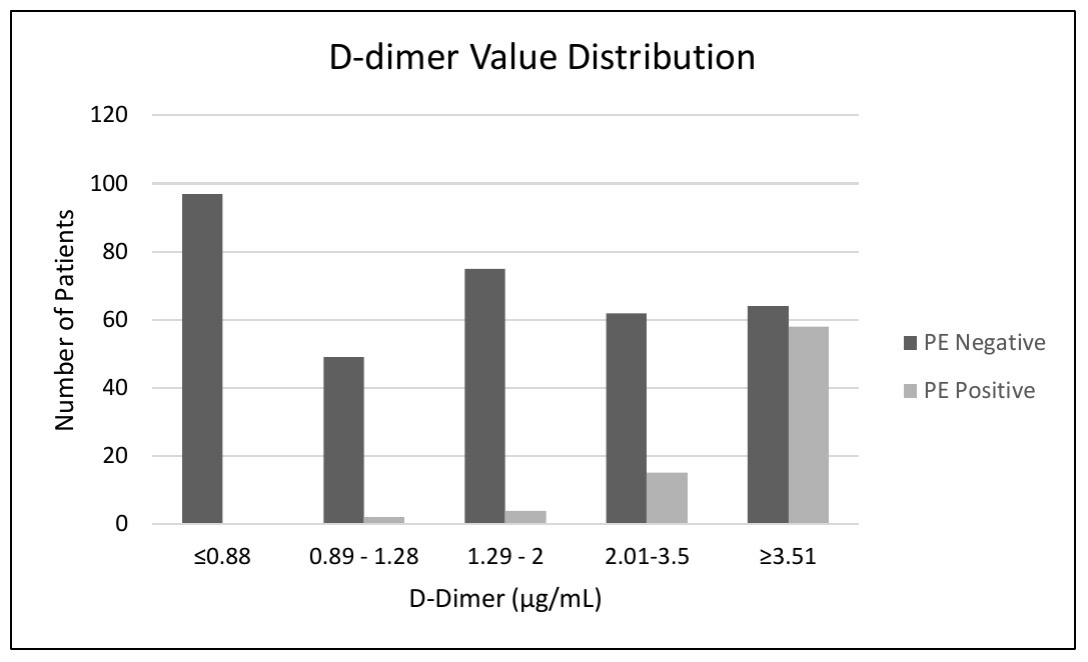
Distribution of D-dimer values between the PE negative and PE positive subgroups.

Applying the traditional ≤ 0.5 μg./mL. D-dimer threshold against our study data yielded a sensitivity for excluding PE to be 100% and specificity of 7.49% (Table 2). The authors were able to increase the D-dimer threshold to < 0.89 μg./mL. to maintain a sensitivity of 100% but raise the specificity to 27.95% (Table 2). Additional analyses of a cut-off value of ≤ 1.28 μg./mL. reduced the sensitivity to 97.47% but increased the specificity to 57.93% (Table 2). Figure 2 depicts the distribution of D-dimer values between the PE negative and PE positive sample subgroups.

**Table 2.** Sensitivity and specificity for pulmonary embolism at specific D-dimer thresholds within our sample population. Includes counts of PE present or absent.

**D-dimer Threshold**		**PE Present**	**PE Absent**	**Sensitivity**	**Specificity**
**≤0.5 μg./mL.**	>0.5 μg./mL.	79	321	**100%**	**7.49%**
	≤0.5 μg./mL.	0	26		
**<0.89 μg./mL.**	≥0.89 μg./mL.	79	250	**100%**	**27.95%**
	<0.89 μg./mL.	0	97		
**≤1.28 μg./mL.**	>1.28 μg./mL.	77	146	**97.46%**	**57.93%**
	≤1.28 μg./mL.	2	201		

PE = Pulmonary Embolism

### Secondary Findings

Additional data were analyzed to draw further conclusions and note trends that may clinically assist providers. Regarding demographics, the mean age of the PE positive group was 61.3 years old (SD 15.6) versus the PE negative group which was 61.7 years old (SD 16.8). This difference was not statistically significant (P = 0.86). Furthermore, of the 79 patients with PE, 42 (53.2%) were male while 37 (46.8%) were female (P = 0.42).

During the peak of COVID-19 at our institution, it became apparent that many of these patients had initial chest X-rays that demonstrated “multifocal pneumonia” but were later found to be positive for PE. In this study, those patients who received a chest X-ray (CXR) (N=407), 321 (78.9%) were identified of having infiltrate in X-ray reports. Those who received pulmonary CT angiography (N=426), 390 (91.6%) were identified to have infiltrate in reports. Although not formally analyzed, these data support the conclusion that infiltrates identified on imaging does not exclude PE in COVID-19 patients.

The authors also compared mortality rates within the analytic sample. Overall, 68 (16.0%) sample patients were deceased at the time of data collection (May-July 2020). The percentage of patients who died in the “positive for PE” group (N=12, 15.2%) versus “negative for PE” group (N=56, 16.1%) showed no significant chi square test difference (P = 0.84), although this may have been attributable to the small sample size.

### Study Limitations

Some typical measurement limitations in COVID-19 study designs include a wide variability in sensitivity of the nasopharyngeal swabs, largely dependent on: a) the types and quality of specimens obtained,^9,10^ b) duration of illness at the time of their testing,^11,12^ and c) the specific assay that was used.^13,14^ This could have led to false negatives which, in turn, would have resulted in missed data points. During the study, we did not take into account certain health conditions (e g., pregnancy, a history of clotting disorder or cancer, ambulatory status, trauma), anticoagulation status, or other co-morbidities such as end stage renal disease and other forms of venous thromboembolism or arterial occlusion which may have affected our results.^15^

Neither did our sample include individuals who underwent alternative diagnostic modalities for venous thromboembolism. As previously mentioned, we also excluded cases in which CT images were read as “inadequate” due to motion artifact (i.e., patient movement) and problematic timing of contrast bolus.

## DISCUSSION

COVID-19 has been cited as causing a pro-inflammatory state which can lead to increased risk for venous thromboembolism.^16^ Based on these results and those from earlier studies, thrombotic complications such as PE are frequently identified in COVID-19 patients and can lead to increased morbidity and mortality if not promptly diagnosed.^2^ The overlap of symptoms has been particularly challenging in determining whom to perform pulmonary CT angiography on. In these analyses, utilization of the D-dimer lab marker may be one tool to assist providers in determining the need for pulmonary CT angiography in COVID-19 patients.

As previously mentioned, the utility of D-dimer lab values to rule out PE has apparently only been validated in non-COVID-19 patients to date.^6^ The traditional D-dimer value of ≤ 0.5 μg./mL. serves as an important tool to rule out PE in patients with a low and intermediate probability for PE. In our n = 426 sample, we were able to maintain a sensitivity of 100% to rule out PE observing a D-dimer threshold of < 0.89 μg./mL while increasing our specificity to 27.95% from 7.49% when compared to the traditional D-dimer threshold. Despite our increase in specificity, the D-dimer still primarily retains its purpose as a negative predictive tool due to the number of aforementioned comorbid conditions that can raise D-dimer levels.^15^

When we observed an increased “< 0.89 ug./mL” D-dimer threshold, we considerably reduced (i.e., approx.. 97 (22.7%) the number of evidently unnecessary pulmonary CT angiography scan orders. In comparison, theoretically applying the traditional D-dimer threshold of ≤ 0.5 μg./mL. to our study only reduced CT scans by 6.1% (i.e., 26 CT scans). This has significant implications for patient safety, cost utilization, and more appropriate use of hospital resources.

During our study, we found a significant difference in average D-dimer value in the PE positive group compared to the PE negative group: 9.15 μg./mL. versus 2.95 μg./mL., respectively. Significantly elevated serum thrombogenic proteins such as the D-dimer have also been associated with increased mortality in COVID-19 patients.^17^ Although the D-dimer serves primarily as a negative predictive tool, providers should be vigilant when significantly elevated D-dimer values are obtained in COVID-19 patients. Although other forms of venous thromboembolism and arterial occlusion were not formally addressed during our study, providers may consider further development of lower D-dimer thresholds for certain patient subgroups receiving additional diagnostic studies.^15^

## CONCLUSIONS

Although thrombotic complications such as PE have been frequently associated with COVID-19, few tools currently exist to identify patients requiring pulmonary CT angiography. Our study findings indicate an increased D-dimer threshold can be reasonably used to rule out PE and eliminate unnecessary pulmonary CT angiography scans.

Prompt diagnosis of PE will generally lead to appropriate therapies and potentially better patient outcomes. Further studies focusing on alternative diagnostic modalities with significantly elevated D-dimer cases are certainly necessary given the associated increased mortality. Further studies considering how empiric anticoagulation may be warranted in clinically unstable COVID-19 patients with elevated D-dimer values are needed.

### Conflicts of Interest

The authors declare no conflicts of interest.
